# The Relationship Between Demographic and Medical Characteristics and the Development of Posttraumatic Stress Disorder in Children Following Emergency Department Attendance: A Prospective Study

**DOI:** 10.1016/j.acepjo.2025.100210

**Published:** 2025-07-08

**Authors:** Anna McKinnon, Zoe Mermin, Tim Dalgleish, Clare Dixon, Andrea Edwards, Richard Meiser-Stedman, Patrick Smith, Devasena Subramanyam, Adrian Boyle

**Affiliations:** 1 McKinnon Psychology, Edgecliff, New South Wales, Australia; 2 Department of Psychology, Princeton, New Jersey, USA; 3 Medical Research Council Cognition and Brain Sciences Unit, University of Cambridge, Cambridge, UK; 4 Sussex Partnership National Health Service Foundation Trust, Sussex, UK; 5 Neonatal Intensive Care Unit, Cambridge University Hospitals NHS Foundation Trust, Cambridge, UK; 6 Department of Clinical Psychology & Psychological Therapies, Norwich Medical School, University of East Anglia, Norwich, UK; 7 Department of Psychology, Institute of Psychiatry, Psychology and Neuroscience, King’s College London, London, UK; 8 Department of Emergency Medicine, Bedford Hospital NHS Trust, Bedford, UK; 9 Department of Emergency Medicine, Cambridge University Hospitals NHS Foundation Trust, Cambridge, UK

**Keywords:** emergency department, post traumatic stress disorder, child, trauma, observational study, violence, victim

## Abstract

**Objectives:**

This study adopted a prospective longitudinal design to assess the utility of demographic and medical characteristics routinely available to emergency medicine clinicians to predict the development of posttraumatic stress disorder (PTSD) in children exposed to death or serious injury 2 months following emergency department (ED) attendance.

**Methods:**

A sample of children (8-17 years; N = 231) were recruited from 4 EDs in the East of England between 2010 and 2013. Within 2 weeks of attendance, research nurses screened records for appropriate cases and recorded information on relevant variables from ED attendance notes. At 2 months, a research assistant carried out a structured clinical interview to assess their Diagnostic and Statistical Manual of Mental Disorders, Fifth Edition (DSM-5) PTSD symptoms. Univariate analyses were conducted to compare ED characteristics between children who developed PTSD and those who did not. Logistic regression models were used to identify variables associated with increased risk of PTSD.

**Results:**

Ten percent of children met the criteria for PTSD at 2 months. Systolic blood pressure, pulse, number of injuries, being subjected to interpersonal violence, and having a head injury were variables that distinguished PTSD and non-PTSD groups. Logistic regression models showed that being assaulted was predictive of PTSD (Odds ratio = 5.07, 95% CI [1.51, 17.00]); although these models had excellent specificity (0.96), the sensitivity was poor (0.30)—that is, there were a number of cases who developed PTSD but were not assaulted.

**Conclusion:**

PTSD is a complication of exposure to death or injury that occurs in a significant minority of children. Children who are victims of interpersonal violence are more likely to develop the disorder.

**The Bottom Line**

This study highlights that while some risk factors can help identify vulnerable children, many cases of posttraumatic stress disorder (PTSD) may still go undetected without broader screening. Therefore, it is important for clinicians to be aware of risk factors for PTSD in children and be aware of the resources available.

## 1 Introduction

### 1.2 Background

An estimated 17 percent of children and adolescents visit an emergency department (ED) in the United States each year.^1^ Posttraumatic stress disorder (PTSD) is a debilitating anxiety disorder,^2^ shown to affect between 12% and 35% of injured children.^3^, ^4^, ^5^, ^6^, ^7^ The disorder is diagnosable 1 month following exposure to a Diagnostic and Statistical Manual of Mental Disorders, Fifth Edition (DSM-5) defined criterion A event: actual or threatened death, serious injury, or sexual violence, hereafter referred to as a criterion A event.^8^ For a diagnosis, a child must endorse symptoms from re-experiencing, avoidance, negative cognition and mood, changes in arousal and reactivity, and experience impairment in functioning.^8^ The disorder can lead to severe and persistent disability and impact upon the child’s entire life course.^9^^,^^10^

Children with more severe injuries usually present through emergency departments, but currently, very few EDs offer advice about identification or responding to the development of PTSD. Identifying children at risk earlier could lead to earlier intervention and recovery. One tool for determining a child’s risk of developing persistent PTSD has been to administer a self-report or structured clinical interview of acute stress disorde.^11^^,^^12^ However, a number of problems with the acute stress disorder diagnosis have been established, particularly in children’s populations.^13^, ^14^, ^15^ Even if these tools had better sensitivity and specificity, they are unlikely to be feasibly deployed in an ED by busy clinicians.

A second approach has been to study demographic-, cognitive-, and injury-related variables associated with increased risk for PTSD. Three meta-analyses^16^, ^17^, ^18^ found small effect sizes for the effects of female gender, criterion A event severity, and previous exposure to a criterion A event. Two other meta-analyses collated studies that investigated the predictive capabilities of variables related to hospital care: Alisic et al^16^ focused on children and Morris et al^19^ included studies involving both children and adults. Both studies found a small effect size for elevated heart rates at ED. Alisic et al^16^ also assessed injury severity and length of hospital stay, finding small effect sizes for both. Many variables have not been examined frequently enough to compute effect sizes; many studies only comprise small samples and the widespread use of brief self-report measures to index PTSD rather than structured interviews with well-established psychometric properties.

### 1.2 Importance

There is inconsistent evidence that medical variables elevate a child’s risk for PTSD, and there is a need for studies in large samples that adopt “gold standard” clinical interviews of PTSD symptoms. Furthermore, studies of criterion A event-exposed children attending ED have mainly used children admitted to hospital, rather than all children who attend ED. This is important, as nonadmitted children represent the majority of all criterion A event-exposed children.^20^ Providing emergency clinicians with evidence-based guidelines as to how to identify children who may need psychological follow-up after discharge may dramatically improve the provision of care for this group.

### 1.3 Goals of This Investigation

The primary aim of this study was to determine whether routinely collected basic demographic characteristics and medical variables (eg, more severe injury), which are collected in the ED, are associated with an increased risk of developing PTSD at 2 months postevent.

## 2 Methods

### 2.1 Study Design and Setting

We conducted a prospective study of children recruited from 4 EDs in East Anglia between September 2010 and April 2013.

### 2.2 Selection of Participants

A member of the clinical care team at each hospital screened the electronic attendance records of all children attending the ED to identify eligible candidates. Inclusion criteria were being exposed to a criterion A event within the previous 2 months. Exclusion criteria included the inability of a child’s parent/caregiver to speak English; intellectual disability; attendance resulting from deliberate self-harm; being under the care of social services or child protection; and moderate-to-severe traumatic brain injury (ie, posttraumatic amnesia ≥ 24 hours). If the person was eligible, the member of the clinical care team recorded the child’s demographics (eg, age and gender) and injury-related data from medical records on a case report form.

The members of the clinical care team invited eligible families by letter. If the family did not opt out of the study within 7 days, a member of the research team was provided with the contact details of the child’s parents. They phoned the family, informing them about the study, answering questions, and seeking their permission to participate. Written, informed consent from both the child and their parent (or primary adult caregiver) was required for participation.

### 2.3 Exposures

The exposure of interest was experiencing a criterion A event preceding ED department attendance. Criterion A events include actual or threatened death, serious injury, or sexual violence.^8^

### 2.4 Measurements and Outcomes

Our measures were extracted from ED records, which included demographics, criterion A event type, injuries, and treatment characteristics.

Our outcome measure, PTSD was assessed with the Children’s Posttraumatic Stress Disorders Inventory-I,^21^ (CPTSD-1) a semistructured clinical interview shown to have excellent psychometrics in previous research. Although the Children’s Posttraumatic Stress Disorders Inventory-I was originally designed to assess DSM-IV diagnostic criteria, additional items were added to assess the revised DSM-5 criteria (Table 1).

**Table 1:** Items added to the CPTSD-I measure.

Question	Corresponding DSM-5 PTSD symptom
Since the event, do you blame yourself for what happened in [EVENT], even though other people say it wasn’t your fault? If "Yes" was indicated, have you been thinking this way for the past week?	Criterion D: persistent, distorted cognitions about the cause or consequences of the traumatic event(s) that lead the individual to blame himself/herself or others.
Since the event, do you blame someone else for what happened in the event, even though other people say it wasn’t their fault? If "Yes" was indicated, have you been thinking this way for the past week?	Criterion D: persistent, distorted cognitions about the cause or consequences of the traumatic event(s) that lead the individual to blame himself/herself or others.
Since the event, do you feel scared a lot of the time? If "Yes" was indicated, have you been feeling this way for the past week?	Criterion D: persistent negative emotional state.
Since the event, do you feel angry a lot of the time? If "Yes" was indicated, have you been feeling this way for the past week?	Criterion D: persistent negative emotional state.
Since the event, do you feel guilty a lot of the time? If "Yes" was indicated, have you been doing this for the past week?	Criterion D: persistent negative emotional state.
Since the event, do you feel ashamed of yourself a lot of the time? If "Yes" was indicated, have you been feeling this way for the past week?	Criterion D: persistent negative emotional state.
Since the event, do you think that the world is a very dangerous place now? If "Yes" was indicated, have you been thinking this way for the past week?	Criterion D: persistent and exaggerated negative beliefs or expectations about oneself, others, or the world.
Since the event, do you think that your life has been ruined by what happened in the event? If "Yes" was indicated, have you been thinking this way for the past week?	Criterion D: persistent and exaggerated negative beliefs or expectations about oneself, others, or the world.
Since the event, do you find it hard to trust other people? If "Yes" was indicated, have you been doing this for the past week?	Criterion D: persistent and exaggerated negative beliefs or expectations about oneself, others, or the world.
Since the event, have you been doing more dangerous things? If "Yes" was indicated, have you been doing this for the past week?	Criterion E: reckless or self-destructive behavior.

CPTSD-1, childhood post traumatic stress disorder-1; DSM, diagnostic statistical manual; PTSD, post traumatic stress disorder.

### 2.5 Data Analysis

All analyses were carried out using SPSS Version 29. We analyzed all available data from ED records to compare characteristics at ED attendance between children who developed PTSD and those who did not, using χ^2^ tests and *t*-tests. To account for age, we also ran a series of logistic regressions with each predictor individually, controlling for age. The contribution of variables to diagnostic status at 2 months was investigated using a logistic regression model. Trauma type was dichotomized as either interpersonal violence (comprising any kind of violence or assault, including dog attack) or noninterpersonal violence. The variables, which differentiated the 2 groups during preliminary analysis, were entered into the model as well as age.

There were substantial amounts of missing data, due in part to the fact that many of the children in this sample had only mild injuries, and therefore, certain data were not collected. Where there were missing data for intubation (missing n = 16; 6.9%) and loss of consciousness (missing n = 20; 8.7%), all analyses were conducted assuming missing values represented a negative endorsement. There was also substantial missing data for the Glasgow Coma Scale (GCS) upon arrival in ED (missing n = 114; 49%) and lowest GCS (missing n = 124; 53.7%). In cases where children did not have a head injury, GCS scores were replaced with the typical functioning of children on that measure (GCS = 15). We replaced these data with values expected of a functioning child as healthy assumptions, as these observations would not have been documented if children presented with only mild injuries.

Of the variables that differentiated the groups, 2 had substantial amounts of missing data: pulse (n = 60; 26%) and systolic blood pressure (missing n = 82; 35.5%). As a sensitivity analysis, the logistic regression was replicated using multiple imputations to account for the missing data. A total of 40 imputed data sets were created; we selected fully conditional specification for the imputation process. To determine whether this was a valid approach, we conducted Little’s missing completely at random (MCAR) test to examine the pattern of missing data for systolic blood pressure and pulse. The test yielded a nonsignificant result (χ^2^ = 3.833, degrees of freedom = 2, *P* = .147), indicating that the missingness for these variables was consistent with the assumption of MCAR. This suggests that the missing values were not systematically related to either observed or unobserved data in the sample.

### 2.6 Ethics Approval

Ethics approval was received from the UK National Research Ethics Service, Cambridgeshire 1 Research Ethics Committee (10/H0304/11) to carry out the study at all 4 sites.

## 3 Results

### 3.1 Characteristics of Subjects

The recruitment flow for the study is summarized in Figure. Invitation letters were sent to the parents of 773 eligible children. The team was unable to make contact with 21% (n = 168) of the families identified; 4% (n = 30) were ineligible, 41% (n = 315) declined participation, and 4% (n = 34) were unreachable for their assessment. Twenty-nine of the 260 families that completed the 2-month PTSD assessment needed to be excluded due to missing data on the key dependent variable of interest.

**Figure:**
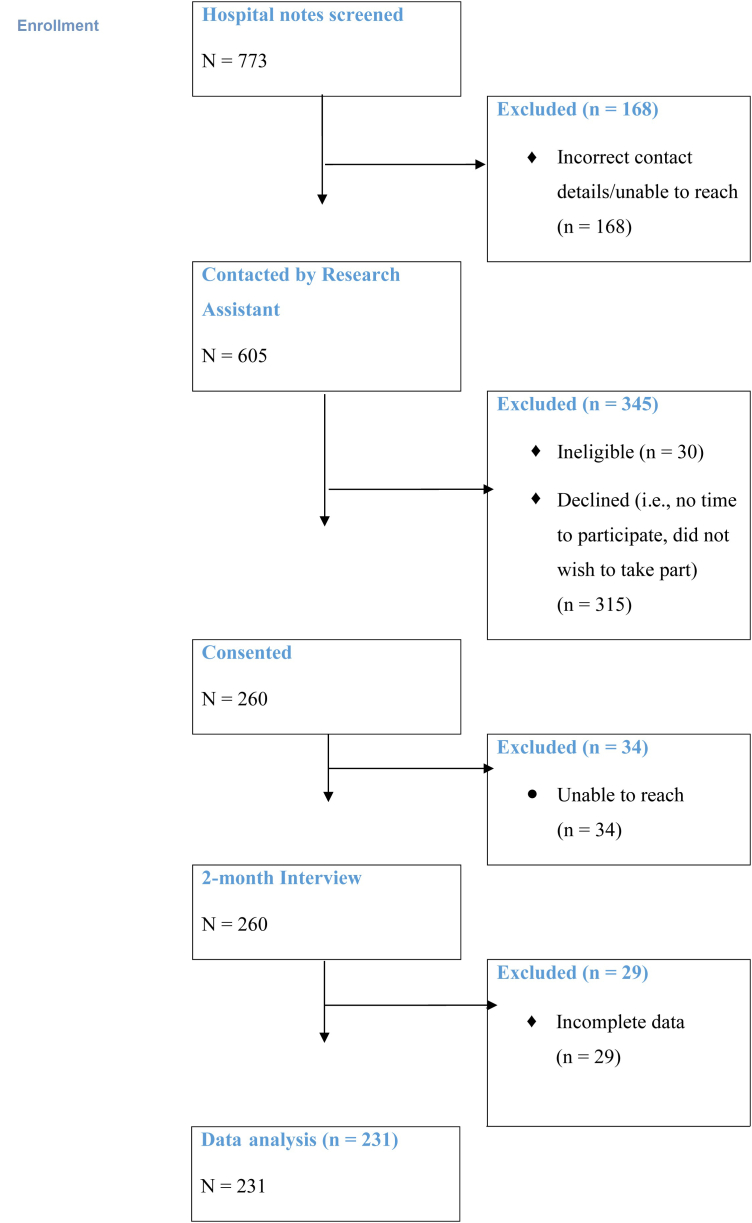
Progression of participants through the study.

When comparing participants (n = 231) with nonparticipants (n = 542), there were no significant differences regarding key demographic or injury-related factors such as age, sex, ethnicity, and several medical observations (all *P* > .05). However, participants were more likely to have a lower GCS rating, been admitted to the hospital, and lost consciousness during their criterion A event. They were also more likely to have been assaulted, received opiate medication at the ED, and been intubated at the scene of the event (all *P* < .03).

Participants were children aged 8-17 years (mean _age_ *=* 14.07 years, SD = 2.93; 57% male). A summary of core demographic and injury-related characteristics in the sample is presented in Table 2. In the final sample, 106 (45.9%) had been involved in a motor vehicle collision; 50 (21.6%) had been subjected to interpersonal violence (12 cases involved a dog attack); 75 (32.5%) had sustained serious accidental injuries; and 1 participant (.4%) had experienced a medical emergency. Participants mostly received mild injuries: 23.4% sustained a fracture, 30.3% were admitted to hospital, 6.5% were admitted to either the pediatric intensive care unit or intensive care unit, and 19.0% received opiate mediations. Notably, 41% received a head injury during the criterion A event, whereas 14.2% lost consciousness during or shortly after the event. Ten (4.7%) were intubated at the scene of the event.

**Table 2:** Demographic, event, injury and treatment characteristics of children with and without PTSD at 2 months.

Variable	No. of participants		Test	Effect size (95% CI)
	No PTSD (n = 208)^a^^,^^b^	PTSD (n = 23)^a^^,^^b^		
Demographic characteristics				
Age, mean (SD), y	13.96 (2.97)	15.04 (2.37)	t(30.2) = −2.02, *P* =.052	g = −0.37 (−0.80, 0.06)
Gender			χ^2^[1] = 0.00, *P* = .949	OR = 1.03 (0.43, 2.45)
Males	119 (57.2)	13 (56.5)		
Female	89 (42.8)	10 (43.5)		
Ethnicity			*P* = 1.000^c^	OR = 0.63 (0.08, 5.02)
White British	194 (93.3)	22 (95.7)		
Ethnic minority	14 (6.7)	1 (4.3)		
Event characteristics				
Type of event^d^			*P* < .001^b^^,^^e^	OR = 6.00 (2.45, 14.74)
Noninterpersonal	171 (82.2)	10 (43.5)		
Interpersonal	37 (17.8)	13 (56.5)		
Arrival method			χ^2^[1] = 1.99, *P* = .158^f^	OR = 1.86 (0.78, 4.43)
Emergency vehicle^g^	120 (57.7)	10 (43.5)		
Private transport	84 (40.4)	13 (56.5)		
Other	2 (1)	0 (0)		
Missing	2 (1)	0 (0)		
Treatment characteristics				
Intubated at the scene			*P* < .604^c^	NA^h^
No	198 (95.2)	23 (100)		
Yes	10 (4.8)	0 (0)		
Admitted to hospital			χ^2^[1] = 2.02, *P* = .156	OR = 0.45 (0.15, 1.38)
No	142 (68.3)	19 (82.6)		
Yes	66 (31.7)	4 (17.4)		
Length of admission, mean (SD), days^i^	3.81 (3.41)	3.00 (1.41)	t(43) = 0.33, *P* = .741	g = 0.24 (−1.16, 1.63)
Admitted to PICU or ICU			*P* = 1.000^c^	OR = 0.63 (0.08, 5.02)
No	194 (93.3)	22 (95.7)		
Yes	14 (6.7)	1 (4.3)		
Seen in the resuscitation room			*P* = .748^c^	OR = 0.66 (0.15, 2.98)
No	180 (86.5)	21 (91.3)		
Yes	26 (12.5)	2 (8.7)		
Missing	2 (1)	0 (0)		
ED attendances in the preceding 12 months, mean (SD)^j^	1.39 (0.75)	1.57 (1.16)	t(229) = −0.10, *P* =.319	g = 0.80 (−0.65, 0.21)
Underwent procedure in ED			χ^2^[1] = 1.86, *P* =.172	OR = 2.34 (0.67, 8.21)
No	52 (25)	3 (13.0)		
Yes	148 (71.2)	20 (87.0)		
Missing	8 (3.8)	0 (0)		
Opiate analgesia given			*P* = .774^c^	OR = 0.65 (0.18, 2.30)
No	160 (76.9)	19 (82.6)		
Yes	39 (18.8)	3 (13)		
Missing	9 (4.3)	1 (4.3)		
Initial ED observations				
Glasgow Coma Scale scores^k^	14.99 (0.11)	14.95 (0.23)	t(18.85) = 0.77, *P* = .449	g = 0.33 (−0.14, 0.81)
Lowest Glasgow Coma Scale score^l^	14.97 (0.17)	14.95 (0.23)	t(182) = 0.52, *P* = .606	g = 0.13 (−0.35, 0.60)
Pulse^m^	84.3 (17.04)	90.1 (16.7)	t(167) = −1.43, *P* =.077	g = −0.34 (−0.81, 0.13)
Systolic blood pressure, mean (SD), mm Hg^n^	118.96 (20.27)	131.35 (12.46)	t(147) = −2.65, *P* = .009^e^	g = −0.63 (−1.11, −0.16)
Diastolic blood pressure, mean (SD), mm Hg^n^	70.35 (10.95)	75.05 (11.16)	t(147) = −1.78, *P* = .077	g = −0.43 (−0.90, 0.05)
Respiratory rate^o^	18.35 (5.24)	18.24 (4.83)	t(139) = 0.09, *P* = .929	g = 0.02 (−0.48, 0.53)
Injury characteristics				
Head injury			χ^2^[1] = 11.76, *P* <.001^e^	OR = 4.85 (1.83, 12.82)
No	130 (62.5)	6 (26.1)		
Yes	76 (36.5)	17 (73.9)		
Missing	2 (1)	0 (0)		
Loss of consciousness			*P* = .194^c^	OR = 2.03 (0.69, 5.96)
No	183 (88)	18 (78.3)		
Yes	25 (12)	5 (21.7)		
No. of injuries, mean (SD)	1.67 (0.87)	2.13 (0.92)	t(229) = −2.39, *P* = .017^p^	g = −0.52 (−0.96, −0.09)
Fracture			χ^2^[1] = 1.86, *P* =.173	OR = 1.88 (0.75, 4.706)
No	162 (77.9)	15 (65.2)		
Yes	46 (22.1)	8 (34.8)		
Self-reported pain score, mean (SD) (0-10)^q^	5.21 (2.66)	6.58 (2.54)	t(137) = −1.72, *P* = .089	g = −0.52 (−1.11, 0.08)

ED, emergency department; ICU, intensive care unit; NA, not applicable; OR, odds ratio; PICU, paediatric intensive care unit; PTSD, post traumatic stress disorder.

a Data are presented as No. (%) unless specified.

b Percentages may not equal 100 due to rounding.

c Inferential statistics calculated using Fisher’s exact test.

d Our definition of interpersonal violence included dog attacks.

e Less than .01.

f Chi-square test conducted with emergency vehicle and private transportation.

g Includes both ambulance arrivals (n = 121) and helicopter arrivals (n = 9).

h Odds ratio could not be calculated given no children with PTSD were intubated.

I n = 45 (no PTSD = 43, PTSD = 2).

j Includes the current visit.

k n = 194 (no PTSD = 175, PTSD = 19).

l n = 184 (no PTSD = 165, PTSD = 19).

m n = 169 (no PTSD = 149, PTSD = 20).

n n = 149 (no PTSD = 129, PTSD = 20).

o n = 141 (no PTSD = 124, PTSD = 17).

p Less than .05.

q n = 139 (no PTSD = 127, PTSD = 12).

### 3.2 Main Results

Twenty-three children (10%) met PTSD diagnosis at 2 months. The demographic and medical/injury characteristics of children with and without PTSD are summarized in Table 2.

Univariate analyses revealed that relative to children without PTSD, those with a PTSD diagnosis at 2 months were more likely to have been subjected to interpersonal violence, have a head injury, have a greater number of injuries, and have a higher systolic blood pressure in ED. There were no significant differences with respect to any other medical and demographic variables (*P* = .052 to .95). This pattern of results remained when controlling for age with 1 exception; pulse became significant (*P* = .025; Table S1).

A logistic regression model was then conducted to assess the relationship between various factors and PTSD. The model, which included interpersonal violence, head injury, number of injuries, systolic blood pressure, pulse, and age, was statistically significant (χ^2^[6] = 35.71, *P* < .001) and explained 39.6% of the variance (Nagelkerke R^2^) in PTSD status at 2 months. Being assaulted was significantly associated with PTSD (*P* = .009, odds ratio = 5.07, 95% CI [1.51, 17.00]). However, the number of injuries, systolic blood pressure, pulse, and head injury were not significantly associated with PTSD (*P* > .05). The model predicted 86.9% of the cases correctly, which was a minor increase of 0.7% on the null model. The model had a specificity of 0.96, but a very low sensitivity of 0.30, that is, it was a strong model for categorizing children as not having PTSD, but poor at categorizing children as having PTSD. The positive predictive value and negative predictive value of the model were 0.55 and 0.90, respectively.

Finally, multiple imputation was used to handle missing data and assess the robustness of the results. The pooled results of the multiple imputation analysis somewhat differed from the results of the main logistic regression. Being assaulted remained significant (*P* = .002, OR = 5.30), whereas pulse (*P* = .034, OR = 1.04) and head injury (*P* = .035, OR = 3.53) became significant. Systolic blood pressure and a number of injuries remained nonsignificant (*P* > .05).

## 4 Limitations

Several limitations should be considered when interpreting the results of this study. First, although the study was longitudinal, there was only 1 follow-up at 2 months; hence, we were unable to determine which variables are associated with a longer-term trajectory of PTSD. This is important because previous work suggests that the pattern of variables predicting PTSD can change over time.^22^ In this case, a longer-term follow-up was not possible, given this cohort was invited to participate in a treatment study.

Second, missing data could introduce potential bias in the findings. Although in our sensitivity analysis, we employed multiple imputation techniques to handle missing data, including systolic blood pressure and pulse, the assumption that missing values were MCAR may not fully capture the complexity of the data. Missing data could still be related to unobserved factors, and thus, our conclusions may not be entirely free from bias.

Additionally, the generalizability of our findings may be limited by the inclusion and exclusion criteria used in the study. Children with moderate-to-severe traumatic brain injury or intellectual disabilities were excluded, which may affect the applicability of the results to these groups. Moreover, the sample was recruited from 4 EDs in East Anglia, UK, and the findings may not be representative of children from other regions or health care settings.

Finally, although this study provides valuable insights into potential risk factors for PTSD in children following trauma, the study’s observational design precludes making causal inferences about the relationships between predictors and PTSD. The findings reflect associations, not causal pathways, and future work should explore these relationships in more depth.

## 5 Discussion

The present study represents the largest trauma-exposed ED child cohort (N = 231) ever studied in Europe. To our knowledge, it is also one of the very few studies to use gold standard structured clinical interview techniques to measure PTSD in children following a criterion A event.

One in 10 children and adolescents met the criteria for PTSD at the follow-up. This study aimed to determine the demographic and medical risk factors associated with the development of PTSD in a large cohort of children recruited from the ED. In the univariate analysis, the following 5 variables were found to be associated with PTSD development at 2 months: having a head injury, number of injuries, pulse and systolic blood pressure at ED, and being the victim of assault. When all 5 variables were entered into a logistic regression controlling for age, only being the victim of interpersonal violence remained significant. This yielded a good model for ruling out nonsymptomatic children at 2 months; however, this was a very poor model for positively identifying children who would later develop PTSD.

Being assaulted was also a significant predictor in the logistic regression using multiple imputations. In addition, pulse and experiencing a head injury were also significant. However, the high levels of missing data for some of the variables (26% for pulse and 36% for systolic) limit their reliability.

Our most robust finding is that interpersonal violence is associated with an increased risk of PTSD, as it was significant in each univariate and multivariate analysis run. This finding is consistent with previous studies, suggesting being assaulted is a risk factor for PTSD development^10^^,^^23^ but does not align with 1 other study in the area.^24^ Our results (from the multivariate analysis using multiple imputation) are consistent with previous meta-analyses in children and adults as well as individual studies of children.^16^^,^^19^^,^^25^, ^26^, ^27^ In this analysis, we also found that receiving a head injury was associated with an increased risk of developing PTSD which is consistent with a recent systematic review that rates of PTSD after traumatic brain injuries were higher than in children with orthopedic injuries.^28^ However, it conflicts with 1 study that found no difference in rates of PTSD of children with and without a mild traumatic brain injury following a motor vehicle collision.^29^ It is important to note that the association between pulse and PTSD and head injuries and PTSD was only present in the logistic regression that imputed a great deal of missing data. Further research on the effect of medical variables such as pulse and head injury with a more robust sample is necessary to determine whether they are accurate predictors of PTSD to which clinicians should pay attention.

We found that 10% of children developed PTSD following a criterion A event, which is slightly lower than estimates found in previous research.^3^, ^4^, ^5^, ^6^, ^7^ One possible reason for this is that we recruited mainly from relatively affluent towns surrounded by a semirural, predominately White population. This is particularly pertinent as assaults are more likely to occur in deprived inner-city environments,^30^ As such, it is not clear whether our results can be generalized to other areas, in particular, deprived inner-city environments.

Our results have important policy implications. They show that there is no need to provide psychological services universally to children within the first few months of a criterion A event, as most children will recover. At the same time, PTSD does affect a significant minority of children involved in a criterion A event, even when their physical injuries are mild. One in 10 children in our sample developed PTSD, and other studies have found higher rates.^3^, ^4^, ^5^, ^6^, ^7^ Therefore, it is important for clinicians to be aware of risk factors for PTSD that children experiencing a criterion A event face. Our results suggest that children suffering interpersonal violence are at a particularly elevated risk of developing the disorder. However, the poor sensitivity suggests that such a marker cannot be used on its own to detect a “high-risk” subgroup with a high probability of developing the disorder, and the needs of children exposed to other forms of trauma should not be ignored. Identification of such a group would likely need to involve the use of PTSD-specific psychological screening tools. Our results suggest that children suffering interpersonal violence are at risk of developing PTSD.

## Author Contributions

AB, TD, RMS, AM, and PS conceived the study and obtained research funding. AB, DS, TD RMS, and AM supervised data collection. AB, AE, CD, AM, and DS undertook recruitment and managed the data, including quality control. AB, AM, and ZM undertook data analysis. AM, ZM, and AB drafted the manuscript, and all authors contributed substantially to its revision. RMS takes responsibility for the paper as a whole.

## Funding and Support

This study was funded through a UK Medical Research Council Clinician Scientist Fellowship awarded to RMS (G0802821). TD was funded by the 10.13039/501100000265UK Medical Research Council. AB was supported by the 10.13039/501100018956NIHR Cambridge Biomedical Research Centre.

## Conflict of Interest

All authors have affirmed they have no conflicts of interest to declare.
