# Supplementary material for: The Relationship Between Demographic and Medical Characteristics and the Development of Posttraumatic Stress Disorder in Children Following Emergency Department Attendance: A Prospective Study
Source: J Am Coll Emerg Physicians Open. 2025 Jul 8;6(4):100210. doi: 10.1016/j.acepjo.2025.100210 (PMC12274928; doi:10.1016/j.acepjo.2025.100210)
Supplement: Table S1 [file mmc1.docx]

**Supplementary Table 1.** Predictors of PTSD in separate logistic regression models, each controlling for Age

| **Variable** | **N** | **p** | **OR (95% CI)** |
| --- | --- | --- | --- |
| *Demographic characteristics* |  |  |  |
| Gender (Male vs Female) | 231 | .949 | 1.03 (0.43, 2.47) |
| Ethnicity (White British vs Ethnic Minority) | 231 | .702 | .67 (0.08, 5.36) |
| *Event characteristics* |  |  |  |
| Type of Event (Interpersonal violence vs Other) ^a^ | 231 | <.001*** | 5.93 (2.40, 14.67) |
| Arrival Method (Private vs Emergency Vehicle) | 227 | .247 | 1.68 (0.70, 4.07) |
| *Treatment characteristics* |  |  |  |
| Intubated at the scene (Yes vs No) | 231 | .999 | NA ^b^ |
| Admitted to hospital (Yes vs No) | 231 | .258 | 0.52 (0.17, 1.62) |
| Length of admission | 45 | .929 | 0.98 (0.55, 1.71) |
| Admitted to PICU or ICU (Yes vs No) | 231 | .754 | 0.72 (0.09, 5.79) |
| Seen in the resuscitation room (Yes vs No) | 229 | .738 | .771 (0.17, 3.54) |
| ED Attendances in the preceding 12 months ^c^ | 231 | .467 | 1.19 (0.75, 1.88) |
| Underwent procedure in ED (Yes vs No) | 223 | .193 | 2.31 (0.66, 8.14) |
| Opiate analgesia given (Yes vs No) | 221 | .561 | 0.69 (0.19, 2.46) |
| *Initial ED observations* |  |  |  |
| GCS scores | 231 | .152 | 0.15 (0.01, 2.02) |
| Lowest GCS | 231 | .481 | 0.44 (0.05, 4.30) |
| Pulse | 169 | .025* | 1.03 (1.00, 1.06) |
| Systolic Blood Pressure | 149 | .022* | 1.04 (1.00, 1.08) |
| Diastolic Blood Pressure | 149 | .088 | 1.04 (0.99, 1.09) |
| Respiratory Rate | 141 | .118 | 1.09 (0.98, 1.22) |
| *Injury characteristics* |  |  |  |
| Head injury (Yes vs No) | 229 | .003** | 4.46 (1.67, 11.89) |
| LOC (Yes vs No) | 231 | .202 | 2.03 (0.69, 6.00) |
| Number of injuries | 231 | .024* | 1.68 (1.07, 2.63) |
| Fracture (Yes vs No) | 231 | .108 | 2.16 (0.85, 5.54) |
| Self-Reported Pain Score (0-10) | 139 | .079 | 1.26 (0.97, 1.63) |

^a^ Our definition of interpersonal violence included dog attacks

^b^ Odds ratio could not be calculated given no children with PTSD were intubated

^c^ Includes the current visit
